# *N*,*N*′-Dibenzyl-2,2′-[(1,3,4-oxadiazole-2,5-di­yl)bis­(*o*-phenyl­eneoxy)]diacet­amide

**DOI:** 10.1107/S1600536808033904

**Published:** 2008-10-31

**Authors:** Wei Wang, Yong Huang, Ning Tang

**Affiliations:** a College of Chemistry and Chemical Engineering, Lanzhou University, Lanzhou 730000, Gansu, People’s Republic of China

## Abstract

The compound, C_32_H_28_N_4_O_5_, which was synthesized by the reaction of 2,5-bis­(2-hydroxy­lphen­yl)-1,3,4-oxadiazole with *N*-benzyl-2-chloro­acetamide, lies on a twofold rotation axis which passes through the mid-point of the N—N bond and the O atom of the oxadiazole unit. The phenyl­ene and oxadiazole rings are almost coplanar [dihedral angle 1.67 (5)°]. The structure is stabilized by intra­molecular N—H⋯O and N—H⋯N hydrogen bonds.

## Related literature

For the biological and physical properties of 1,3,4-oxadiazole derivatives, see Gómez-Saiz *et al.* (2002 ▶); Wen *et al.* (2003 ▶); Kuo *et al.* (2006 ▶). For literature on metal complexes, see Dong *et al.* (2003 ▶); Zhou *et al.* (1996 ▶).
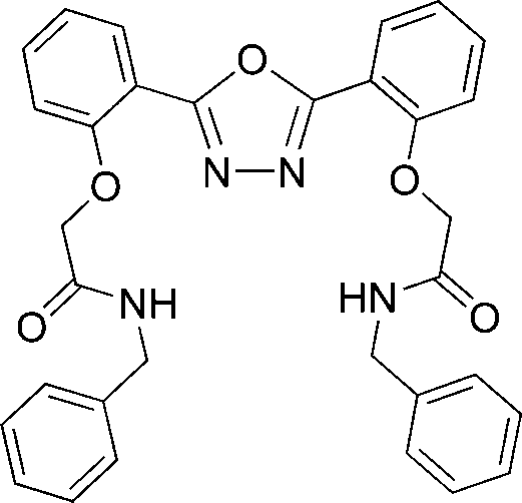


## Experimental

### 

#### Crystal data

C_32_H_28_N_4_O_5_
*M* *_r_* = 548.58
Monoclinic, 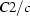

*a* = 17.0619 (15) Å
*b* = 15.2601 (15) Å
*c* = 10.6555 (9) Å
β = 90.611 (5)°
*V* = 2774.2 (4) Å^3^
*Z* = 4
Mo *K*α radiation
μ = 0.09 mm^−1^
*T* = 293 (2) K
0.53 × 0.40 × 0.30 mm

#### Data collection

Bruker APEXII diffractometer
Absorption correction: none
7913 measured reflections
2880 independent reflections
2335 reflections with *I* > 2σ(*I*)
*R*_int_ = 0.022

#### Refinement

*R*[*F* ^2^ > 2σ(*F* ^2^)] = 0.041
*wR*(*F* ^2^) = 0.158
*S* = 1.17
2880 reflections
187 parameters
H-atom parameters constrained
Δρ_max_ = 0.18 e Å^−3^
Δρ_min_ = −0.13 e Å^−3^

### 

Data collection: *APEX2* (Bruker, 2001 ▶); cell refinement: *SAINT* (Bruker, 2001 ▶); data reduction: *SAINT*; program(s) used to solve structure: *SHELXS97* (Sheldrick, 2008 ▶); program(s) used to refine structure: *SHELXL97* (Sheldrick, 2008 ▶); molecular graphics: *SHELXTL* (Sheldrick, 2008 ▶); software used to prepare material for publication: *SHELXTL*.

## Supplementary Material

Crystal structure: contains datablocks I, global. DOI: 10.1107/S1600536808033904/ng2497sup1.cif

Structure factors: contains datablocks I. DOI: 10.1107/S1600536808033904/ng2497Isup2.hkl

Additional supplementary materials:  crystallographic information; 3D view; checkCIF report

## Figures and Tables

**Table 1:** Hydrogen-bond geometry (Å, °)

*D*—H⋯*A*	*D*—H	H⋯*A*	*D*⋯*A*	*D*—H⋯*A*
N2—H2*A*⋯O2	0.86	2.15	2.5523 (16)	109
N2—H2*A*⋯N1	0.86	2.49	3.3524 (17)	177

**Fig. 1.**
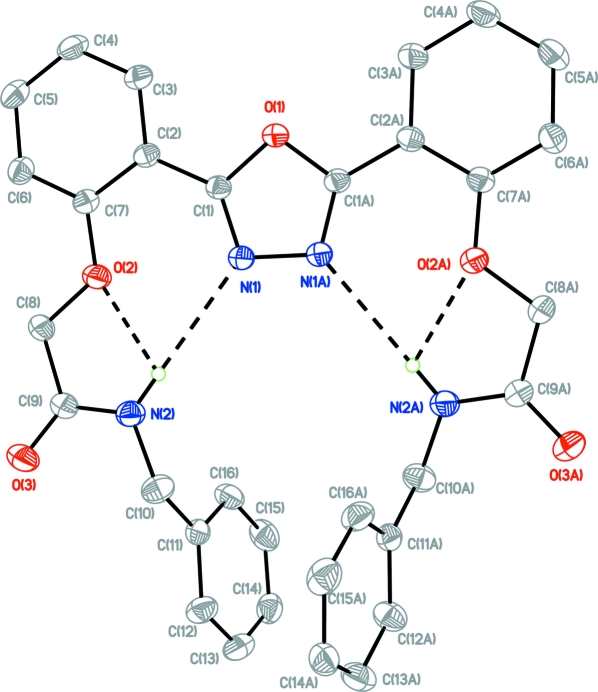
The structure of the title compound, showing 20% probability displacement ellipsoids and the atom-labelling scheme. The intramolecular hydrogen bonds are shown as dashed lines.

## supplementary crystallographic information

### Comment

Heterocyclic 1,3,4-oxadiazole and its derivatives have been studied for a long time because many of these derivatives show biological activity (Patricia *et al.*, 2002) and electron-transporting capability (Wen *et al.*, 2003), in particular as active compounds in organic light emitting diodes (OLEDs) (Kuo *et al.*, 2006). At the same time, these five-membered oxadiazole ring can bind metal ions by N and O donors to expand various novel polymeric frameworks, some with open channels and interesting luminescence properties (Dong *et al.*, 2003). We herein report the synthesis and crystal structure of a new amide-based 1,3,4-oxadiazole bridging ligand, namely the title compound, (I).

As seen from Fig. 1, the molecule of (I) possesses crystallographically imposed C_2_ symmetry, with the two fold axis bisecting the central 1,3,4-oxadiazole ring. The central two phenyl rings and oxadiazole ring are almost coplanar. The amide groups of molecule appear to form intramolecular hydrogen bonds with both the phenoxy O atom and the oxadiazole N atom (Table 1). In addition, the centroid-to-centroid distance (4.344 Å) of the two terminal benzene rings is so much longer that it is difficult to regard this as representing a significantly π-π stacking interaction.

### Experimental

To 2,5-bis[2'-hydroxyl-phenyl]-1,3,4-oxadiazole (Zhou *et al.*, 1996) (1.78 g, 7 mmol) in DMF (80 ml) was added sodium hydroxide (0.56 g, 14 mmol). The mixture was heated to 353 K and stirred for about 1 h. A solution of *N*-benzyl-2-chloroacetamide (2.94 g, 16 mmol) and potassium iodide (0.83 g, 5 mmol) in DMF (20 ml) was then added dropwise at a constant rate over 1 h. The reaction mixture was stirred at *ca* 353 K for an additional 48 h. The solvent was removed under vacuum, and then the residue was treated with water (100 ml). The precipitate was collected by filtration and washed with water (100 ml), then twice recrystallized from methanol to give colourless block crystals. ^1^H NMR (400*M* Hz; CDCl_3_, δ, p.p.m): 9.19 (*t*, 2H, N—H, *J* = 6 Hz), 8.04 (*d*, 2H, Ar—H, *J* = 8 Hz), 7.58 (*t*, 2H, Ar—H, *J* = 8 Hz), 7.24–7.15 (*m*, 12H, Ar—H), 7.06 (*d*, 2H, Ar—H, *J* = 8 Hz), 4.72 (*s*, 4H, O—CH_2_), 4.32 (*d*, 4H, Ar—CH_2_—N, *J*= 6 Hz); Yield 2.30 g (60%); m.p. 462–464 K; elemental analysis, calculated for C_32_H_28_N_4_O_5_, C 70.06, H 5.14, N 10.21%; found: C 70.18, H 5.02, N 10.22%. Colourless single crystals suitable for an X-ray diffraction study were obtained by slow evaporation of the methanol solvent at room temperature over a period of 5 d.

### Refinement

All H atoms were initially located in a difference Fourier map and refined freely along with an isotropic displacement parameter. H atoms were positioned geometrically and treated as riding, with C—H = 0.93 and 0.97%A, N—H = 0.86%A,and *U*_iso_(H) = 1.2*U*_eq_(C,*N*).

### Crystal data

**Table:**

C_32_H_28_N_4_O_5_	*F*(000) = 1152
*M**_r_* = 548.58	*D*_x_ = 1.313 Mg m^−^^3^
Monoclinic, *C*2/*c*	Melting point: 462 K
Hall symbol: -C2yc	Mo *K*α radiation, λ = 0.71073 Å
*a* = 17.0619 (15) Å	Cell parameters from 3319 reflections
*b* = 15.2601 (15) Å	θ = 2.4–27.5°
*c* = 10.6555 (9) Å	µ = 0.09 mm^−^^1^
β = 90.611 (5)°	*T* = 293 K
*V* = 2774.2 (4) Å^3^	Block, colourless
*Z* = 4	0.53 × 0.40 × 0.30 mm

### Data collection

**Table:**

Bruker APEXII diffractometer	2335 reflections with *I* > 2σ(*I*)
Radiation source: sealed tube	*R*_int_ = 0.022
graphite	θ_max_ = 26.5°, θ_min_ = 1.8°
φ and ω scans	*h* = −21→20
7913 measured reflections	*k* = −12→19
2880 independent reflections	*l* = −13→12

### Refinement

**Table:**

Refinement on *F*^2^	Secondary atom site location: difference Fourier map
Least-squares matrix: full	Hydrogen site location: inferred from neighbouring sites
*R*[*F*^2^ > 2σ(*F*^2^)] = 0.041	H-atom parameters constrained
*wR*(*F*^2^) = 0.158	*w* = 1/[σ^2^(*F*_o_^2^) + (0.1*P*)^2^] where *P* = (*F*_o_^2^ + 2*F*_c_^2^)/3
*S* = 1.17	(Δ/σ)_max_ = 0.001
2880 reflections	Δρ_max_ = 0.18 e Å^−^^3^
187 parameters	Δρ_min_ = −0.13 e Å^−^^3^
0 restraints	Extinction correction: SHELXL97 (Sheldrick, 2008), Fc^*^=kFc[1+0.001xFc^2^λ^3^/sin(2θ)]^-1/4^
Primary atom site location: structure-invariant direct methods	Extinction coefficient: 0.0054 (10)

### Special details

**Table:**

Geometry. All e.s.d.'s (except the e.s.d. in the dihedral angle between two l.s. planes) are estimated using the full covariance matrix. The cell e.s.d.'s are taken into account individually in the estimation of e.s.d.'s in distances, angles and torsion angles; correlations between e.s.d.'s in cell parameters are only used when they are defined by crystal symmetry. An approximate (isotropic) treatment of cell e.s.d.'s is used for estimating e.s.d.'s involving l.s. planes.
Refinement. Refinement of *F*^2^ against ALL reflections. The weighted *R*-factor *wR* and goodness of fit *S* are based on *F*^2^, conventional *R*-factors *R* are based on *F*, with *F* set to zero for negative *F*^2^. The threshold expression of *F*^2^ > σ(*F*^2^) is used only for calculating *R*-factors(gt) *etc*. and is not relevant to the choice of reflections for refinement. *R*-factors based on *F*^2^ are statistically about twice as large as those based on *F*, and *R*- factors based on ALL data will be even larger.

### Fractional atomic coordinates and isotropic or equivalent isotropic displacement parameters (Å^2^)

**Table:**

	*x*	*y*	*z*	*U*_iso_*/*U*_eq_	
O1	0.5000	1.06829 (8)	0.7500	0.0488 (3)	
O2	0.37163 (7)	0.91715 (7)	0.50427 (11)	0.0724 (4)	
O3	0.32801 (7)	0.70855 (8)	0.38437 (12)	0.0822 (4)	
N1	0.47305 (7)	0.93150 (7)	0.69949 (10)	0.0494 (3)	
N2	0.41511 (7)	0.75799 (8)	0.52944 (11)	0.0586 (3)	
H2A	0.4317	0.8025	0.5714	0.070*	
C1	0.45891 (7)	1.01308 (8)	0.67330 (11)	0.0443 (3)	
C2	0.40733 (7)	1.05489 (9)	0.58052 (11)	0.0466 (3)	
C3	0.40137 (9)	1.14557 (10)	0.57514 (13)	0.0580 (4)	
H3A	0.4307	1.1796	0.6308	0.070*	
C4	0.35278 (9)	1.18603 (10)	0.48861 (15)	0.0677 (4)	
H4A	0.3493	1.2468	0.4865	0.081*	
C5	0.30953 (9)	1.13641 (11)	0.40562 (15)	0.0650 (4)	
H5A	0.2772	1.1639	0.3469	0.078*	
C6	0.31367 (8)	1.04601 (10)	0.40866 (14)	0.0582 (4)	
H6A	0.2839	1.0127	0.3528	0.070*	
C7	0.36253 (7)	1.00528 (9)	0.49553 (11)	0.0496 (3)	
C8	0.32734 (8)	0.86010 (9)	0.42649 (13)	0.0560 (4)	
H8A	0.3328	0.8770	0.3392	0.067*	
H8B	0.2723	0.8633	0.4480	0.067*	
C9	0.35711 (8)	0.76851 (9)	0.44569 (13)	0.0558 (4)	
C10	0.45065 (9)	0.67305 (10)	0.55103 (16)	0.0654 (4)	
H10A	0.5040	0.6820	0.5809	0.078*	
H10B	0.4534	0.6424	0.4714	0.078*	
C11	0.40855 (7)	0.61462 (8)	0.64410 (12)	0.0508 (3)	
C12	0.41917 (10)	0.52501 (10)	0.63923 (16)	0.0682 (4)	
H12A	0.4500	0.5013	0.5762	0.082*	
C13	0.38524 (10)	0.46989 (11)	0.72545 (16)	0.0738 (5)	
H13A	0.3937	0.4098	0.7209	0.089*	
C14	0.33913 (10)	0.50362 (12)	0.81753 (14)	0.0709 (5)	
H14A	0.3168	0.4668	0.8768	0.085*	
C15	0.32603 (11)	0.59234 (12)	0.82183 (15)	0.0785 (5)	
H15A	0.2937	0.6156	0.8832	0.094*	
C16	0.36072 (10)	0.64747 (10)	0.73521 (14)	0.0666 (4)	
H16A	0.3514	0.7075	0.7390	0.080*	

### Atomic displacement parameters (Å^2^)

**Table:**

	*U*^11^	*U*^22^	*U*^33^	*U*^12^	*U*^13^	*U*^23^
O1	0.0557 (7)	0.0377 (7)	0.0528 (7)	0.000	−0.0038 (6)	0.000
O2	0.0861 (8)	0.0440 (6)	0.0862 (8)	0.0010 (5)	−0.0371 (6)	−0.0052 (5)
O3	0.0976 (9)	0.0571 (8)	0.0913 (8)	−0.0035 (6)	−0.0217 (7)	−0.0175 (6)
N1	0.0536 (6)	0.0409 (6)	0.0537 (6)	−0.0004 (5)	−0.0055 (5)	−0.0002 (4)
N2	0.0599 (7)	0.0475 (7)	0.0682 (8)	−0.0027 (5)	−0.0049 (6)	0.0025 (5)
C1	0.0460 (6)	0.0409 (7)	0.0461 (7)	−0.0030 (5)	0.0036 (5)	−0.0002 (5)
C2	0.0466 (7)	0.0435 (7)	0.0496 (7)	−0.0019 (5)	0.0049 (5)	0.0055 (5)
C3	0.0656 (8)	0.0450 (8)	0.0632 (9)	−0.0054 (6)	−0.0017 (7)	0.0075 (6)
C4	0.0776 (10)	0.0459 (8)	0.0794 (11)	0.0010 (7)	−0.0042 (8)	0.0185 (7)
C5	0.0630 (9)	0.0619 (10)	0.0699 (9)	0.0028 (7)	−0.0057 (7)	0.0227 (7)
C6	0.0556 (8)	0.0609 (9)	0.0581 (8)	−0.0002 (6)	−0.0055 (6)	0.0075 (6)
C7	0.0506 (7)	0.0446 (7)	0.0536 (7)	−0.0002 (6)	0.0006 (6)	0.0047 (5)
C8	0.0567 (7)	0.0526 (9)	0.0584 (8)	−0.0057 (6)	−0.0091 (6)	−0.0043 (6)
C9	0.0578 (8)	0.0503 (8)	0.0593 (8)	−0.0043 (6)	0.0004 (6)	−0.0044 (6)
C10	0.0572 (8)	0.0597 (10)	0.0794 (10)	0.0068 (7)	0.0053 (7)	0.0083 (7)
C11	0.0485 (7)	0.0522 (8)	0.0516 (7)	0.0032 (6)	−0.0073 (5)	−0.0003 (5)
C12	0.0724 (10)	0.0589 (9)	0.0734 (10)	0.0188 (7)	0.0086 (8)	0.0065 (7)
C13	0.0816 (11)	0.0548 (9)	0.0850 (12)	0.0083 (8)	−0.0040 (9)	0.0156 (8)
C14	0.0796 (10)	0.0749 (11)	0.0581 (9)	−0.0146 (9)	−0.0069 (8)	0.0125 (7)
C15	0.0893 (12)	0.0861 (13)	0.0603 (10)	−0.0136 (10)	0.0152 (9)	−0.0130 (8)
C16	0.0819 (10)	0.0534 (8)	0.0644 (9)	−0.0039 (7)	0.0056 (7)	−0.0143 (7)

### Geometric parameters (Å, °)

**Table:**

O1—C1	1.3627 (14)	C6—C7	1.3863 (18)
O1—C1^i^	1.3627 (14)	C6—H6A	0.9300
O2—C7	1.3570 (16)	C8—C9	1.500 (2)
O2—C8	1.4151 (16)	C8—H8A	0.9700
O3—C9	1.2262 (17)	C8—H8B	0.9700
N1—C1	1.2979 (16)	C10—C11	1.5197 (19)
N1—N1^i^	1.408 (2)	C10—H10A	0.9700
N2—C9	1.3352 (19)	C10—H10B	0.9700
N2—C10	1.4484 (19)	C11—C16	1.3699 (19)
N2—H2A	0.8600	C11—C12	1.3805 (19)
C1—C2	1.4630 (16)	C12—C13	1.378 (2)
C2—C3	1.389 (2)	C12—H12A	0.9300
C2—C7	1.4009 (17)	C13—C14	1.365 (2)
C3—C4	1.379 (2)	C13—H13A	0.9300
C3—H3A	0.9300	C14—C15	1.373 (2)
C4—C5	1.373 (2)	C14—H14A	0.9300
C4—H4A	0.9300	C15—C16	1.386 (2)
C5—C6	1.382 (2)	C15—H15A	0.9300
C5—H5A	0.9300	C16—H16A	0.9300
			
C1—O1—C1^i^	103.62 (13)	O2—C8—H8B	110.0
C7—O2—C8	120.64 (11)	C9—C8—H8B	110.0
C1—N1—N1^i^	106.43 (7)	H8A—C8—H8B	108.4
C9—N2—C10	121.32 (13)	O3—C9—N2	124.01 (14)
C9—N2—H2A	119.3	O3—C9—C8	119.20 (14)
C10—N2—H2A	119.3	N2—C9—C8	116.78 (12)
N1—C1—O1	111.76 (11)	N2—C10—C11	115.39 (11)
N1—C1—C2	132.28 (12)	N2—C10—H10A	108.4
O1—C1—C2	115.95 (11)	C11—C10—H10A	108.4
C3—C2—C7	118.20 (12)	N2—C10—H10B	108.4
C3—C2—C1	120.38 (12)	C11—C10—H10B	108.4
C7—C2—C1	121.42 (12)	H10A—C10—H10B	107.5
C4—C3—C2	121.12 (14)	C16—C11—C12	117.94 (13)
C4—C3—H3A	119.4	C16—C11—C10	122.48 (13)
C2—C3—H3A	119.4	C12—C11—C10	119.57 (12)
C5—C4—C3	119.91 (15)	C13—C12—C11	121.56 (14)
C5—C4—H4A	120.0	C13—C12—H12A	119.2
C3—C4—H4A	120.0	C11—C12—H12A	119.2
C4—C5—C6	120.56 (14)	C14—C13—C12	119.91 (16)
C4—C5—H5A	119.7	C14—C13—H13A	120.0
C6—C5—H5A	119.7	C12—C13—H13A	120.0
C5—C6—C7	119.57 (14)	C13—C14—C15	119.41 (14)
C5—C6—H6A	120.2	C13—C14—H14A	120.3
C7—C6—H6A	120.2	C15—C14—H14A	120.3
O2—C7—C6	123.90 (12)	C14—C15—C16	120.36 (15)
O2—C7—C2	115.47 (11)	C14—C15—H15A	119.8
C6—C7—C2	120.63 (13)	C16—C15—H15A	119.8
O2—C8—C9	108.39 (12)	C11—C16—C15	120.77 (15)
O2—C8—H8A	110.0	C11—C16—H16A	119.6
C9—C8—H8A	110.0	C15—C16—H16A	119.6

Symmetry codes: (i) −*x*+1, *y*, −*z*+3/2.

### Hydrogen-bond geometry (Å, °)

**Table:**

*D*—H···*A*	*D*—H	H···*A*	*D*···*A*	*D*—H···*A*
N2—H2A···O2	0.86	2.15	2.5523 (16)	109
N2—H2A···N1	0.86	2.49	3.3524 (17)	177
